# Long-term effects of the gait treatment using a wearable cyborg hybrid assistive limb in a patient with spinal and bulbar muscular atrophy: a case report with 5 years of follow-up

**DOI:** 10.3389/fneur.2023.1143820

**Published:** 2023-06-08

**Authors:** Kensuke Iijima, Hiroki Watanabe, Yuichi Nakashiro, Yuki Iida, Michio Nonaka, Fumio Moriwaka, Shinsuke Hamada

**Affiliations:** ^1^Department of Rehabilitation, Hokuyukai Neurological Hospital, Sapporo, Japan; ^2^Department of Neurosurgery, Institute of Medicine, University of Tsukuba, Tsukuba, Japan; ^3^Department of Neurology, Hokuyukai Neurological Hospital, Sapporo, Japan

**Keywords:** spinal and bulbar muscular atrophy, wearable cyborg hybrid assistive limb (HAL), gait treatment, walking symmetry, walking endurance

## Abstract

**Background:**

Spinal and bulbar muscular atrophy (SBMA) is a progressive neuromuscular degenerative disease characterized by the degeneration of lower motor neurons in the spinal cord and brainstem and neurogenic atrophy of the skeletal muscle. Although the short-term effectiveness of gait treatment using a wearable cyborg hybrid assistive limb (HAL) has been demonstrated for the rehabilitation of patients with SBMA, the long-term effects of this treatment are unclear. Thus, this study aimed to investigate the long-term effects of the continued gait treatment with HAL in a patient with SBMA.

**Results:**

A 68-year-old man with SBMA had lower limb muscle weakness and atrophy, gait asymmetry, and decreased walking endurance. The patient performed nine courses of HAL gait treatment (as one course three times per week for 3 weeks, totaling nine times) for ~5 years. The patient performed HAL gait treatment to improve gait symmetry and endurance. A physical therapist adjusted HAL based on the gait analysis and physical function of the patient. Outcome measurements, such as 2-min walking distance (2MWD), 10-meter walking test (maximal walking speed, step length, cadence, and gait symmetry), muscle strength, Revised Amyotrophic Lateral Sclerosis Functional Assessment Scale (ALSFRS-R), and patient-reported outcomes, were evaluated immediately before and after gait treatment with HAL for each course. 2MWD improved from 94 m to 101.8 m, and the ALSFRS-R gait items remained unchanged (score 3) for approximately 5 years. The patient could maintain walking ability in terms of gait symmetry, walking endurance, and independence walking despite disease progression during HAL treatment.

**Conclusion:**

The long-term gait treatment with HAL in a patient with SBMA may contribute to the maintenance and improvement of the gait endurance and ability to perform activities of daily living. The cybernics treatment using HAL may enable patients to relearn correct gait movements. The gait analysis and physical function assessment by a physical therapist might be important to maximize the benefits of HAL treatment.

## Introduction

Spinal and bulbar muscular atrophy (SBMA) is a progressive neuromuscular degenerative disease characterized by the degeneration of lower motor neurons in the spinal cord and brainstem and neurogenic atrophy of the skeletal muscle (1). SBMA occurs only in adult men (1). The typical symptoms are muscle weakness and atrophy, mainly in the proximal parts of the limbs and ball paralysis (2, 3). Sensory deficits may localize to the distal lower extremities (4). The phenomenon of fibrous bundle contraction is another characteristic finding during the voluntary contraction of the facial and neck muscles (1). The age of SBMA onset is 30–60 years. In most cases, the tremors and painful muscle spasms in the fingers precede the disease onset (5). There was no recovery for SBMA. The disease follows a slowly progressive course, usually requiring a wheelchair for mobility 10–15 years after onset. Respiratory failure is a common cause of death by bulbar paralysis (5). However, the kind of rehabilitation that is effective for patients with SBMA is not clear. A progressive neuromuscular disease places a considerable burden on muscles even with mild exercise (6). For SBMA rehabilitation, it is very difficult to set the amount, frequency, and type of exercise. Thus, there are only a few reports on effective rehabilitation methods for SBMA (7, 8).

The main treatment method of rehabilitation for patients with SBMA is symptomatic therapy (9). Leuprorelin—which inhibits the nuclear transportation of testosterone and abnormal androgen receptors—was clinically demonstrated to improve dysphagia in patients with SBMA (10). However, leuprorelin use was not associated with improvements in ambulatory function (10). Several clinical trials of gait treatment using the wearable cyborg hybrid assistive limb (HAL) have been conducted in patients with neurological problems (11–13). Recently, a physician-initiated clinical trial (NCY-3001 study) on gait treatment with HAL for eight rare diseases, including SBMA, was conducted (14). The NCY-3001 study validated the efficacy and safety of HAL gait treatment for an intractable neuromuscular disease with gait disturbance (14). Gait treatment with HAL has resulted in improved gait ability and balance performance (14). In the NCY-3001 study, HAL gait treatment showed significant improvements in the 2-min walking distance (2MWD), cadence at the 10-meter walking test (10MWT), and total scores of the manual muscle testing (MMT) compared with conventional methods (14). The effectiveness of HAL gait treatment for intractable neuromuscular diseases was proved by this doctor-initiated randomized controlled trial (14).

However, the NCY-3001 study was a short-term randomized crossover trial with nine sessions, up to four per week. Some points were unclear about the effects of HAL gait treatment. Moreover, there are no reports on the long-term efficacy and safety of the gait treatment with HAL and appropriate treatment intervals. In addition, previous studies have shown improvements in 2MWD, cadence at 10MWT, and muscle strength (14), but the effects on other outcomes are unclear, such as gait symmetry, activities of daily living (ADL), and treatment satisfaction of the patient, such as the Japanese version of the Decision Regret Scale (DRS).

We had the opportunity to continue HAL gait treatment in a patient with SBMA with reduced walking endurance for approximately 5 years. Thus, this study aimed to report the results of the long-term effects of HAL gait treatment on a patient with SBMA.

## Materials and methods

### Patient

**Figure 1:**
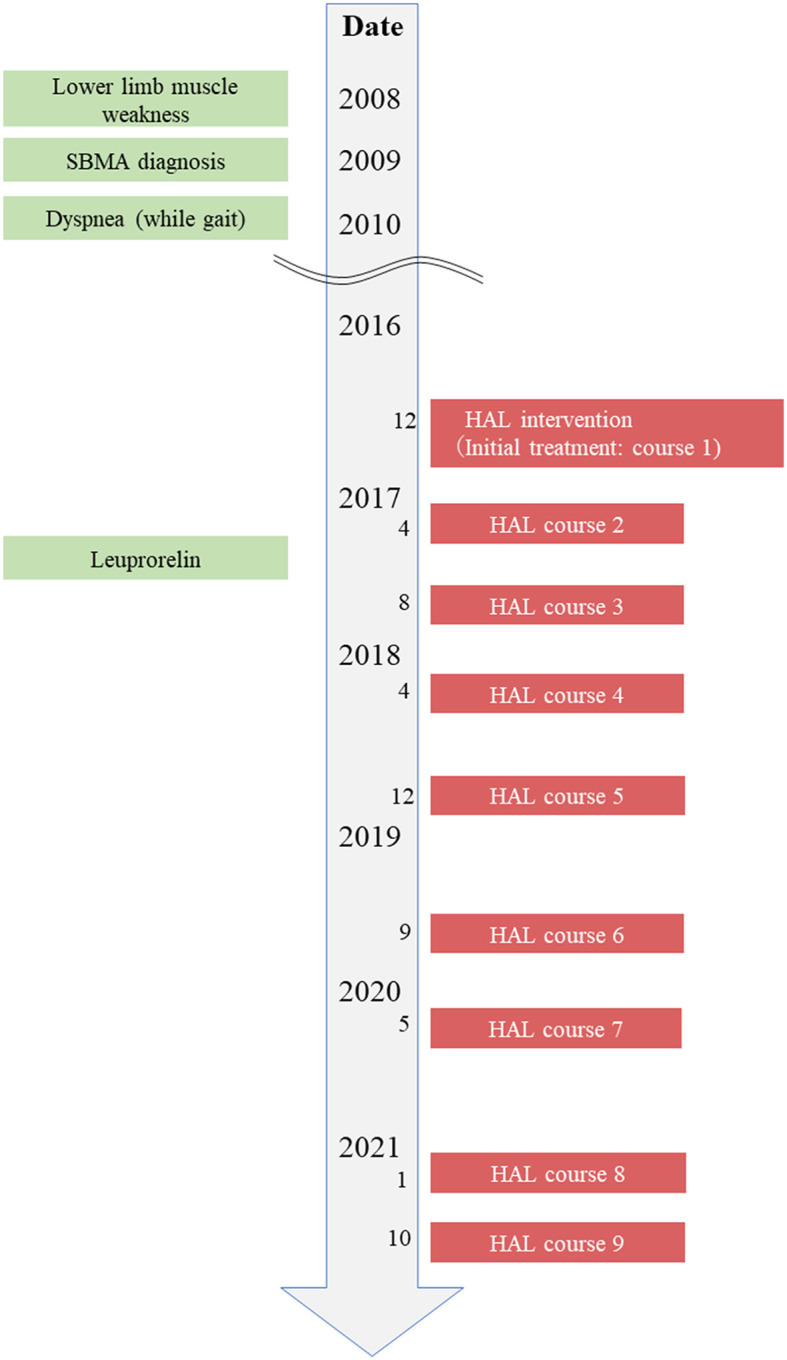
Timeline of interventions. Timeline of patient's history, diagnostic process, and interventions.

The patient was a 68-year-old man who lived with his wife. He was able to perform independently all his ADL before the first course of HAL gait treatment. The patient was not employed, and before SBMA onset, his hobby was going to the museums, which influenced him to draw pictures. The chief complaint of the patient was difficulty walking with the right foot and feeling of fatigue when walking long distances. He hoped to visit museums again using public transportation. The patient wanted to walk more than 500 m without resting, and he wanted to appreciate the museum environment. His height, weight, and body mass index were 168 cm, 55.6 kg, and 20.1 kg/m^2^, respectively. As regards his current medical history, the patient had finger tremors at the age of 35 years and painful swelling of the mammary gland since the age of 40. The patient has decreased muscle strength in the right lower extremity from the age of 60, and an abnormal creatine kinase (CK) value was discovered at a nearby clinic. The CAG repeat count of the patient was abnormal at 46 in the Kennedy genetic analysis at the same years; subsequently, the patient was diagnosed with SBMA. He began to experience dyspnea when walking at the age of 62 and became aware of the progression of muscle weakness in the lower limbs (right > left) from the age of 63. The patient has used an ankle-foot orthosis (Gait Solution-Design^®^: GSD, Pacific Supply, Japan) when walking. He had decreased muscle strength in the upper extremities from the age of 66 and has started regular rehabilitation hospitalization in our hospital for gait treatment with HAL from the age of 68 years (December 2016) (Figure 1). The patient has started leuprorelin acetate once every 12 weeks from November 2017 (ongoing). Regarding his family history, his father has been diagnosed with spinal muscular atrophy and his nephew with SBMA. Medical histories included hypertension and diabetes mellitus.

The neurological findings of the patient before HAL treatment (first course) are as follows: he had a clear level of consciousness and good communication ability. The score of the Japanese version of the Montreal Cognitive Assessment was 28/30. In the MMT, the muscle strength of the lower limbs (right/left) was as follows: hip flexion, 3/4; hip extension, 3/4; knee flexion, 3/4; knee extension, 4/4; ankle dorsiflexion (DF), 4/4; and plantarflexion (PF), 2-/2. The patient had no limitations in the range of motion (ROM); however, there was concave deformation in both legs. The superficial sensation in the sole decreased in the right side dominance, and warm pain and position sensation decreased in the right peripheral limb. The patient's bilateral patellar tendon reflex and Achilles tendon reflex disappeared. The patient had muscle atrophy of the tongue and right lower limb.

The gait ability, gait posture, and gait treatment policy of the patient before HAL treatment (first course) are as follows. The patient was able to walk independently using a Lofstrand crutch in the right upper limb and wearing a GSD in the right lower limb. The patient showed a slight trunk forward tilt in the mid-stance (MSt) to the terminal stance (TSt) on the right side, and the hip extension motion reached initial contact (IC) on the left side without reaching the extension range. Thereafter, the hip joint heeled off in a mildly flexed position in the right TSt to pre-swing (PSw), and the load shifted to the left (Supplementary Figure S1). The stance time during walking was shorter on the right side than on the left side, and the swing time was shorter on the left side. Consequently, the patient showed a temporal asymmetric gait. The continuous walking distance of the patient was approximately 200 m. When the patient walked more than 150 m, dyspnea occurred, and the muscle spasms of the right lower limb appeared at 200 m. The results showed that the patient has insufficient endurance for walking. A previous study reported that asymmetric ambulatory activity negatively affected energy cost during walking (15). The inefficient gait caused by the shortening of the right-side stance phase perhaps caused the decreased walking endurance. The aim of this treatment using HAL was to acquire an efficient gait by the expansion of the hip joint extension motion in the right-side stance phase and accordingly improve the walking endurance to realize the patient's desire.

### Setting and methods of HAL gait treatment

The method of HAL gait treatment was based on a previous study (NCY-3001 study) (14). Gait treatment with HAL was conducted three times per week for 3 weeks, totaling nine times as one course. Fitting was mainly performed according to the HAL-medical leg-type proper-use guide in the initial treatment of each course (16). Fitting was performed by the responsible physical therapist so that the patients received the appropriate assistance. The physical therapist selected the cybernic voluntary control (CVC) mode in all sessions. The CVC means that the assisting torque controls the movement based on the intensity of the bioelectric signals (BES) (16, 17). BES are motor unit potentials on the skin; they correspond to the motor torque required for each joint movement, including the hip and knee, in accordance with the wearer's motor intention (14). Moreover, the physical therapist adjusted the torque tuner and balance tuner based on the alignment, walking observation (gait posture), and body function. The torque tuner indicates the strength of the assistance provided by the four motors at the right and left hip and knee joints, adjustable in 21 levels at each joint (0–20). The balance tuner indicates the balance between the flexion and extension movements of the hip and knee joints and is adjustable in 20 levels at each joint (flexion, FX1–10; or extension, EX1–10). For example, if a physical therapist wants to increase the hip extension assist in a patient's supporting leg, the physical therapist adjusts the balance tuner by one step in the extension dominance (EX1). The patient performed the gait treatment with HAL for approximately 60 min, including setting up, wearing HAL, and break times. The physical therapist selected the task (STAND or WALK) according to the treatment that the patient wanted to perform. The physical therapist selected a task from five levels (WALK 1–5) according to the patient's walking speed. The walking speed is a guide, and the physical therapist adjusted it according to the patient's comfort level when walking faster or slower. In this study, WALK 5 was selected in all sessions. The patient used an all-in-one walker (All in One^®^, Ropox A/S, Denmark) with an unloading function for fall prevention during the treatment, and the physical therapist measured blood pressure and pulse rate before and after the intervention. The intensity of HAL gait treatment was set to the patient's fatigue level that did not exceed “slightly tight” using the modified Borg score (18). The physical therapist checked the presence or absence of muscular fatigue after treatment and on the next day. The walking distance and time during HAL gait treatment were recorded for each course between December 2016 and October 2021.

### Conventional physical therapy

The conventional physical therapy was performed for 40–60 min 3–6 days per week during hospitalization. It included ROM practice, sensory input to the sole, muscle force enhancement training, balance practice, and walking practice. The walking intensity was defined as the distance the patient could walk continuously without rest and was only recorded during courses 1–6.

### Outcome measurements

#### Gait evaluation

Treatment outcome measures were evaluated immediately before and after HAL gait treatment for each course. The primary outcome was 2MWD, and the secondary outcomes were 10MWT (maximal walking speed, step length, and cadence) and muscle strength as measured by MMT. Notably, 2MWD measured before initiating course 1 was compared with that measured after completing course 9 to clarify the long-term effects of HAL treatment. Moreover, we evaluated 10MWT using a portable three-axis accelerometer (Mimamori-Gait ^®^, LSI Medience Corp, Japan) to determine walking speed, step length, cadence, and swing times of both legs. Furthermore, the symmetry index (SI) was calculated using the left–right swing times to evaluate the detailed symmetry during gait (19) as follows:


$$\begin{array}{l} \text{Symmetry}\;\text{Index}\;\left(\text{SI}\right)\\ =\frac{\text{Left}\;\text{swing}\;\text{times}\;-\;\text{Right}\;\text{swing}\;\text{times}}{1/2\left(\text{Left}\;\text{swing}\;\text{times}\;+\;\text{Right}\;\text{swing}\;\text{times}\right)}\times \;100 \end{array}$$

The swing time indicates the values for each swing time on both legs in the 10-m interval.

If the SI is close to 0, the left and right swing times during walking are equal; that is, walking with bilaterally symmetric leg movements is realized. A negative value also represents a shorter swing time on the left than on the right.

#### Muscle strength using MMT

The MMT total score (0–60) was calculated by summing the scores of each lower limb (bilateral flexion and extension of hip, knee, and ankle) (14).

#### Severity of SBMA using the revised amyotrophic lateral sclerosis functional assessment scale (ALSFRS-R) and serum creatinine levels

A previous study conducted by Hasizume et al. assessed the severity of SBMA using the ALSFRS-R (ALS-functional rating scale) and serum creatinine levels (mg/dL) (20). The ALSFRS-R was developed as a comprehensive severity index for patients with ALS and consists of four parts: bulbar function, ADL, respiratory status, and upper and lower extremities (21). A previous study suggested that the serum creatinine level is the most useful blood parameter to detect the severity of motor dysfunction in SBMA. This study also revealed a strong positive correlation between the serum creatinine level and clinical parameters, such as ALSFRS, grip power, and 6-min walking distance (6MWD) at baseline (20). Therefore, we measured the ALSFRS-R and serum creatinine as disease indices of SBMA. Both the ALSFRS-R and serum creatinine level were calculated for the annual decline rate based on the previous studies: [(follow-up data) – (baseline data)/observational period (years)] (20).

#### Patient-reported outcomes (PRO) using the Japanese version of the DRS

The Japanese version of the DRS analyzes patients' feelings of loss of expectations after treatment; thus, we measured the effects of long-term HAL use using this scale after course 9. The DRS is a self-administered rating scale; there were five question items, and responses are scored using a Likert scale, ranging from 1 (strongly agree) to 5 (strongly disagree), with scale scores ranging from 0 to 100. Positive questions (questions 1, 3, and 5) are drawn 1 from the response number, whereas negative questions (questions 2 and 4) are drawn 5 to the response number, and the total score of five questions is multiplied by 5 to calculate the scale score. The higher the score, the higher the decision regret (22).

## Results

### Change in the primary outcome measure (2MWD)

**Figure 2:**
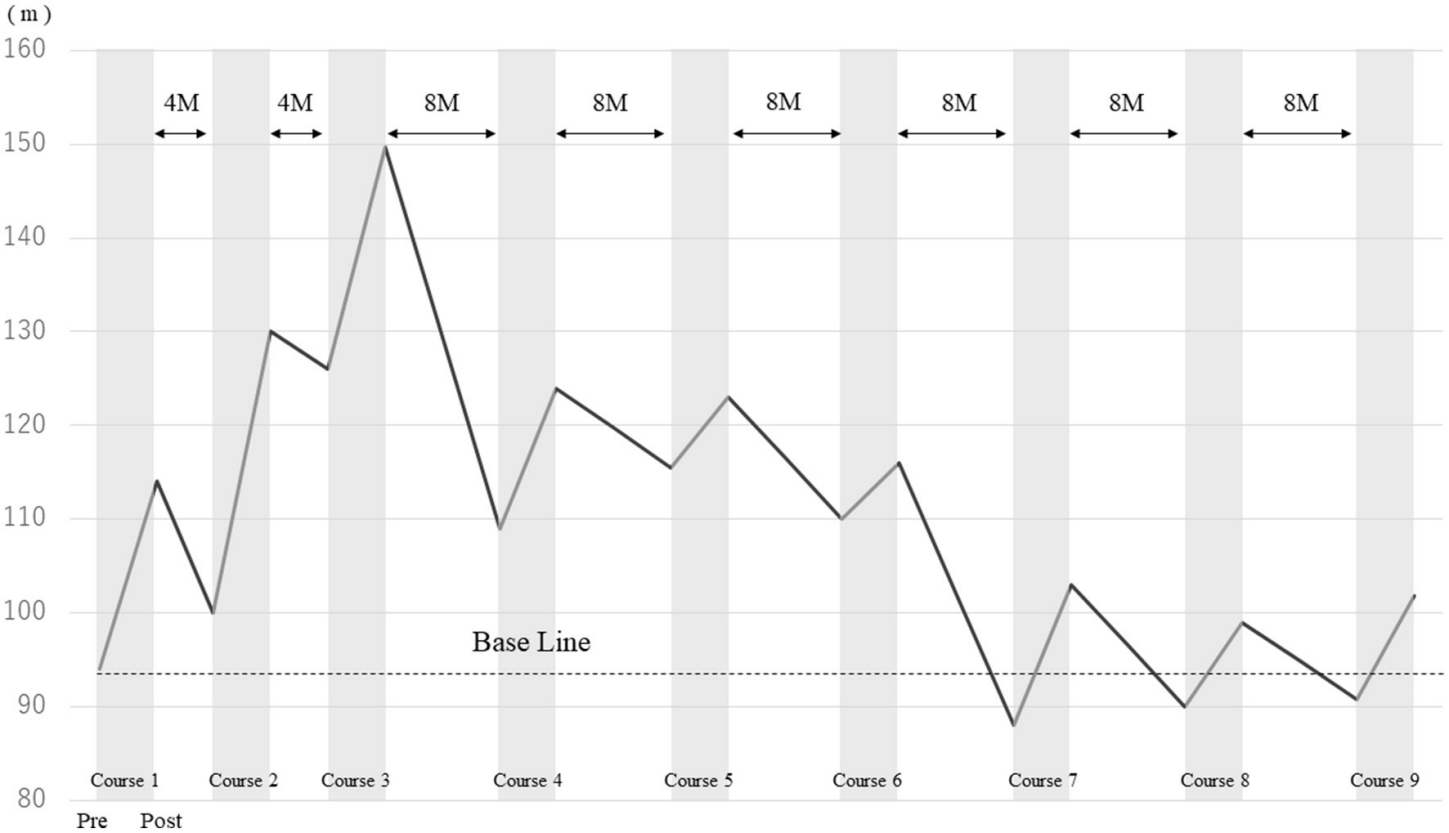
Comparison of the 2-min walking distance before and after HAL gait treatment in each course.

2MWD improved in each course after HAL gait treatment (Figure 2). The improvement rates of 2MWD for each course were as follows: course 1, +21.1%; course 2, +30.0%; course 3, +18.8%; course 4, +13.8%; course 5, +6.5%; course 6, +5.5%; course 7, +17.0%; course 8, +10.0%; and course 9, +12.1%. The result of 2MWD was less than the baseline for the first time in course 7 before the assessment. Thereafter, 2MWD in course 8 and course 9 pre-assessments were also less than the baseline; however, all were higher after HAL treatment. The improvement rate of 2MWD was +8.3% compared with course 1 pre-initiation and course 9 termination, showing an improvement in 2MWD within 5 years.

### Changes in walking capacity and HAL adjustments accordingly to the gait posture

The maximal walking speed, step length, cadence, and SI were improved pre- and post-HAL treatment for each course (Table 1). The patient's condition had worsened daily, therefore the physical therapist adjusted HAL assistance according to the gait posture of the patient during the 5 years of HAL gait treatment. Detailed changes for the gait posture and adjustments for HAL assistance are as follows:

**Table 1:** Change in the walking ability and total muscle strength before and after HAL gait treatment in each course.

	**Course 1**		**Course 2**		**Course 3**		**Course 4**		**Course 5**		**Course 6**		**Course 7**		**Course 8**		**Course 9**	
	**Pre**	**Post**	**Pre**	**Post**	**Pre**	**Post**	**Pre**	**Post**	**Pre**	**Post**	**Pre**	**Post**	**Pre**	**Post**	**Pre**	**Post**	**Pre**	**Post**
Velocity (m/s)	0.87	1.07	1.08	1.17	1.15	1.23	1.10	1.12	0.98	1.14	0.90	1.08	0.60	0.89	0.70	0.73	0.86	0.96
Cadence (steps/min)	1.79	1.93	1.93	2.02	1.99	2.08	2.00	2.03	1.87	2.03	1.85	1.95	1.60	1.71	1.50	1.45	1.82	1.92
Step length (cm)	49.0	55.6	54.0	58.3	58.0	59.3	55.3	55.3	52.3	56.0	48.6	55.6	42.0	53.6	47.0	50.0	47.6	51.2
Rt step time (s)	0.59	0.51	0.56	0.49	0.49	0.48	0.50	0.49	0.56	0.50	0.53	0.54	0.60	0.58	0.63	0.65	0.64	0.62
Lt step time (s)	0.52	0.51	0.56	0.50	0.50	0.48	0.49	0.49	0.49	0.49	0.50	0.52	0.65	0.61	0.70	0.72	0.71	0.68
Symmetry index	−12.6	0.0	0.0	2.0	2.0	0.0	−2.0	0.0	−13.3	−2.0	−5.8	−3.8	8.0	5.0	10.5	10.2	10.4	9.2
Muscle score (total)	40	40	42	42	42	42	42	42	41	41	41	41	39	39	39	39	36	36

#### Course 1

The mild trunk forward-leaning of the patient was observed in MSt to TSt on the right side before the initiation of HAL gait treatment and hip extension movements in TSt reached IC on the left side without reaching the extension range of the hip. The right hip joint remained in a mildly flexed position from TSt to PSw, and in that state, weight-bearing was shifted to the left leg (Supplementary Figure S1A). The swing time during walking was 0.52 s on the left side, whereas it was 0.59 s on the right side. The swing time on the left side was short. Regarding the HAL setting, the physical therapist adjusted the torque tuner of the right hip joint to 2 and the balance tuner to EX5. The EX5 of the balance tuner means that the motion assistance of HAL provided more support on hip extension than on hip flexion. The physical therapist visually checked the symmetry of the gait of the patient and adjusted the motion assistance from HAL to bring the patient's gait closer to normal. The patient could keep in the mid-trunk position from the right MSt to TSt at course 1. The motion of the right hip extension was expanded in the right stance phase, and the motion of the left hip flexion was expanded in the left swing phase (Supplementary Figure S1B). The swing times of both legs were 0.51 s, and the SI changed from −12.6 to 0. The patients acquired symmetric gait after HAL treatment.

#### Courses 2–4

The walking ability of the patient was maintained after HAL treatment for course 1, and no significant side-to-side differences were found in the left and right swing times before and after HAL treatments for courses 2–4. The balance tuner was changed to EX1.

#### Course 5

The right hip extension movement decreased from the right MSt to the TSt, and the swing time of the left leg decreased. The SI at this time was −13.3, and the side-to-side difference was recognized. The balance tuner was set to EX3. The motion of the right hip extension in the right stance phase was expanded, and the SI improved to 2 after the gait treatment with HAL for course 5.

#### Courses 7–9

Regarding the disease progression, muscle weakness around the left hip (Table 1), bilateral sensory impairment, and progression of muscular atrophy were observed before HAL treatment for courses 7–9. As a result, the patient had knee joint instability in the left stance phase, shortening the left-side stance time, causing asymmetric gait, and lowering the endurance. The torque tuner was increased to EX2 of the left hip joint, and the balance tuner was changed in the extension dominance of both the hip joint (EX1) and knee joint (EX1) to compensate for the instability in the knee joint.

### Walking distance of the conventional physical therapy and HAL gait treatment

In conventional physical therapy, the average walking distance was 350.9 m from course 1 to course 6. During gait treatment with HAL, the average walking distance was 1,441.2 m from course 1 to course 6. Moreover, the average walking distance was 1,574.6 m from course 1 to course 9. No adverse events, such as falls or fractures, were noted during the 5-year follow-up period.

### Progression of SBMA using the ALSFRS-R and serum creatinine

Table 2 shows changes in the ALSFRS-R score from course 1 to course 9. The annual percentage change in the total score of ALSFRS-R over the 5 years was −1.6. ALSFRS-R items, such as salivation, handwriting, cutting food, handing utensils, dressing, hygiene, turning in bed and adjusting bedclothes, climbing stairs, and dyspnea were gradually decreased. However, there was no change in swallowing, speech, walking, orthopnea, and respiratory insufficiency. The serum creatinine level was 0.36 mg/dL before course 1 and 0.40 mg/dL after course 9, and the 5-year annual percentage change was +0.008.

**Table 2:** Change in the ALSFRS-R score before and after HAL gait treatment in each course.

	**Course 1**		**Course 2**		**Course 3**		**Course 4**		**Course 5**		**Course 6**		**Course 7**		**Course 8**		**Course 9**	
	**Pre**	**Post**	**Pre**	**Post**	**Pre**	**Post**	**Pre**	**Post**	**Pre**	**Post**	**Pre**	**Post**	**Pre**	**Post**	**Pre**	**Post**	**Pre**	**Post**
1. Speech	4	4	4	4	4	4	4	4	4	4	4	4	4	4	4	4	4	4
2. Salivation	4	4	4	4	4	4	4	4	4	4	4	4	3	3	3	3	3	3
3. Swallowing	2	2	2	2	2	2	2	2	2	2	2	2	2	2	2	2	2	2
4. Handwriting	4	4	4	4	4	4	4	4	4	4	4	4	4	4	3	3	3	3
5. Cutting food and handling utensils	4	4	4	4	4	4	4	4	4	4	4	4	3	3	3	3	3	3
6. Dressing and hygiene	4	4	4	4	4	4	3	3	3	3	3	3	3	3	3	3	3	3
7. Turning in bed and adjusting bed clothes	4	4	4	4	4	4	3	3	3	3	3	3	3	3	3	3	3	3
8. Walking	3	3	3	3	3	3	3	3	3	3	3	3	3	3	3	3	3	3
9. Climbing stairs	3	3	3	3	3	3	2	2	2	2	2	2	1	1	1	1	1	1
10. Dyspnea	4	4	4	4	3	3	3	3	3	3	3	3	3	3	3	3	3	3
11. Orthopnea	4	4	4	4	4	4	4	4	4	4	4	4	4	4	4	4	4	4
12. Respiratory insufficiency	4	4	4	4	4	4	4	4	4	4	4	4	4	4	4	4	4	4
Total	44	44	44	44	43	43	40	40	40	40	40	40	37	37	36	36	36	36

ALSFRS-R, revised amyotrophic lateral sclerosis functional assessment scale.

### PRO using the Japanese version of the DRS

The patient answered the following changed points: “the body became straight and gait was stabilized”, “the feeling of walking was realized by kicking the ground”, “the step length increased”, and “the feeling of fatigue decreased and muscle cramps decreased” after HAL gait treatment for course 1. In daily life, the patient answered “increased frequency of going out” and “decreased frequency of rests when going out”. The DRS score after course 9 was 5 points. In the DRS questionnaire, the patient stated: “If I had not done HAL treatments, I could not walk in daily living now. Therefore, I have no regrets about HAL gait treatment (high satisfaction)”. Regarding HAL treatment, the patient commented, “The time required to wear HAL is too long. As I had to stand while wearing HAL, I already felt a little fatigued before starting the HAL treatment”.

## Discussion

This case report aimed to determine the long-term effects of HAL gait treatment on gait disorders in a patient with SBMA. Based on the results of the present study, the patient exhibited improved short-term gait symmetry and endurance as well as maintained long-term gait endurance and gait ability for ADL and muscle mass. The results also suggest that the continuous long-term use of HAL in patients with SBMA can help them to maintain their walking ability. In a previous report of a patient with SBMA, the gait function of the patient was improved and maintained without damaging the motor unit, and it was reported that combining leuprorelin with cybernic treatment (i.e., using gait treatment with HAL) may suppress disease progression (23). As reported in the results of the present study, the average walking distance during gait treatment with HAL was approximately 1,500 m per session, which is four times greater than that for conventional gait training without HAL. Moreover, the patient with SBMA could walk long distances without experiencing excessive fatigue. Although we could not report the changes in CK levels over time in the present report, the use of HAL may have prevented the overloading of the remaining muscle tissues as suggested in the previous study (23), thereby resulting in an adequate amount of walking exercise without excessive fatigue.

### Short-term effects of HAL gait treatment

This study indicated that the patient with SBMA had improved walking endurance, gait velocity, step length, and gait symmetry after HAL gait treatment in each course. The cybernic treatment with HAL may have been successful as a contributing factor to these results (14, 17). The physical function and gait analysis by a physical therapist before the gait treatment with HAL are essential for cybernic treatment. The physical therapist confirmed decreased superficial and deep sensations in the periphery of the lower extremity and muscle strength in the right lower extremity periphery before HAL treatment (course 1). Therefore, the patient lacked sensory information associated with load shifting during walking.

The extension motion of the hip joint of the patient in MSt to TSt was insufficient because of the lowering muscle strength of the right leg and lowering supportiveness by the hypoesthesia of the periphery. Furthermore, the shortening of the stance phase on the right side of the patient resulted in the shortening of the swing time on the left side; thus, the patient learned incorrectly (mislearning) the asymmetric gait on the over-ground walking without HAL. The physical therapist adjusted the torque tuner of the right hip joint to 2, and the balance tuner was set to hip extension dominance (EX5). Moreover, the physical therapist performed the gait treatment with HAL appropriately while visually checking the gait symmetry. The patient had decreased muscle strength of the left hip joint, bilateral sensory disturbance, and progression of muscular atrophy in both legs in course 7. The worsening muscle atrophy resulted in knee joint instability in the left stance; thus, a shortening of the left-side stance time caused gait asymmetry and decreased walking endurance. The physical therapist increased the amount of torque tuner of the left hip joint and left knee joint, and the balance tuner was set in the extension dominance of both the hip and knee joints. The patient could walk without knee joint instability during the gait treatment with HAL. During the study, the physical therapist recognized decreased physical function, such as decreased muscle strength, muscle atrophy, sensory disturbance and gait ability walking endurance, and walking symmetry. The gait analysis by the physical therapist revealed that the gait and posture based on the evaluation of the physical functions and the use of a wearable sensor indicate that the patient could more comfortably walk each time using HAL. As a result, the patient had improved walking endurance and walking symmetry after HAL gait treatment in every course. A previous study reported that asymmetric ambulatory activity negatively affected the energy costs during walking (15). Therefore, HAL gait treatment (cybernic treatment) may have contributed to the improvement in walking endurance after the patient developed a symmetric gait.

### Long-term effects of HAL gait treatment

This study suggested that the long-term continuous use of HAL can maintain the walking ability of patients with SBMA. It was found that 2MWD, ALSFRS-R gait items, and serum creatinine of the patient after courses 1 and 9 of HAL treatment were similar to those at the baseline assessment. In the relevant literature, few studies have reported the natural course of SBMA, which has been found to progress gradually, eventually leading to the need for a wheelchair for mobility. Notably, Atsuta et al. (5) conducted a longitudinal observational study of 223 Japanese patients with SBMA (55.2 ± 10.5 years) to elucidate the natural course of SBMA based on the ADL milestones; they reported that the average time interval between the onset of muscle weakness and becoming wheelchair-bound was 13.2 ± 7.8 years in patients with SBMA and CAG repeat counts of ≤ 47 (5). However, our patient could still independently walk 13 years after the onset of SMBA. Hashizume et al. investigated the natural course of the disease in 34 Japanese patients with genetically confirmed SBMA in terms of the annual rate of decline for each endpoint over 3 years (20). They found that the yearly declines in 6MWD, ALSFRS-R, and serum creatinine levels were −20.3 ± 26.0 m, −1.1 ± 0.9, and −0.013 ± 0.03 mg/dL, respectively. 6MWD has been considered the most reliable measure of motor impairment in SBMA owing to its ability to reliably detect a 10% decline in 1 year (24, 25). A previous study reported a strong correlation between 6MWD and 2MWD in patients with neuromuscular disease (26). We measured 2MWD instead of 6MWD in the present study, and we observed an improvement in 2MWD after each HAL treatment; moreover, our patient maintained the achieved 2MWD for over 3 years. Although not directly comparable, 2MWD was expected to decrease by −6.76 ± 8.66 m per year according to a previous study (20). 2MWD was 94 m, the ALSFRS-R score was 44 points, and the serum creatinine level was 0.36 mg/dL before course 1 of HAL treatment. The predicted 5-year prognosis at this time was 2MWD of 60.2 m, ALSFRS-R of 38.5, and serum creatinine level of 0.29 mg/dL based on a previous study (20). In this study, 2MWD was 101.8 m, the ALSFRS-R was 36 (no reduction in walking items), and the serum creatinine was 0.40 mg/dL after course 9 of HAL gait treatment. 2MWD and serum creatinine levels were good compared with the natural course of SBMA.

The total MMT score decreased from 40 to 36 in 5 years. The lower extremity muscle strength decreased in both hip flexion, left hip extension, and both ankle PF and maintained in the right hip extension, both knee extension, and both ankle DF. The ALSFRS-R score decreased beyond prognostic prediction, and in this patient, disease progression may be relatively rapid compared with the natural disease course. However, the score of the gait items in the ALSFRS-R did not change. In addition, the serum creatinine values increased compared with baseline assessment. In general, serum creatinine is associated with whole muscle mass (20). Our results suggested that HAL gait treatment might contribute to improvement in walking endurance and progression of muscle atrophy. Long-term HAL gait treatment for patients with SBMA may maintain and improve gait endurance and gait ability for daily activities; therefore, it may also contribute to the maintenance of muscle mass. In addition, the results of the DRS after course 9 showed that the patient had no regrets (high satisfaction) with HAL gait treatment. HAL gait treatment provided long-term satisfaction for the patient with SBMA.

### Study limitations

First, the appropriate interval of the use of HAL gait treatment cannot be mentioned. 2MWD increased progressively during courses 1–3; HAL gait treatment was conducted at 4-month intervals, and the longest 2MWD was reached after course 3. On the contrary, 2MWD gradually shortened after course 4. Indeed, HAL gait treatment was conducted at 8-month intervals after course 3. The decline in walking ability due to the natural disease course is also in the background; however, the effect of HAL gait treatment may change depending on the treatment interval. We think that there is a need to shorten HAL treatment interval as much as possible. Furthermore, the benefits of HAL treatment at 8-month intervals should be carefully assessed. Second, the results of this study are limited to a single case and include limiting factors, such as the ABA design and lack of a control group with respect to treatment intervals. Finally, the effects of conventional physical therapy and medication other than HAL gait treatment may become a confounding factor in terms of the long-term effectiveness of HAL gait treatment. Despite these limitations, only a few reports have evaluated the long-term effects of HAL gait treatment on patients with SBMA. Our results may help researchers set the protocol and outcomes of HAL gait treatment for patients with SBMA. The long-term effects of HAL gait treatment should be evaluated in more individuals with SBMA in the future.

## Conclusion

Continuous HAL gait treatment might inhibit disease progression only in terms of mobility items in ADL, walking endurance, and overall body muscle mass in patients with SBMA. The correct HAL settings based on the appropriate evaluation by the physical therapist may be important for efficient HAL gait treatment. We need to reconsider the pretreatment evaluation method for the correct settings in HAL gait treatment and shorten HAL treatment interval as much as possible.

## Data availability statement

The original contributions presented in the study are included in the article/Supplementary material, further inquiries can be directed to the corresponding author.

## Ethics statement

Ethical review and approval was not required for the study on human participants in accordance with the local legislation and institutional requirements. The patient/participant provided their written informed consent to participate in this study. Written informed consent was obtained from the individual for the publication of any potentially identifiable images or data included in this article.

## Author contributions

KI and HW were the major contributors to manuscript writing and literature review. KI administered HAL treatment and collected, analyzed, and interpreted the data. YN, YI, MN, FM, and SH provided valuable comments about the case report. All authors revised the manuscript and approved the final version.
