# Supplementary material for: Long-term effects of the gait treatment using a wearable cyborg hybrid assistive limb in a patient with spinal and bulbar muscular atrophy: a case report with 5 years of follow-up
Source: Front Neurol. 2023 Jun 8;14:1143820. doi: 10.3389/fneur.2023.1143820 (PMC10285061; doi:10.3389/fneur.2023.1143820)
Supplement: Supplementary file 1 [file Data_Sheet_1.docx]

Supplementary Material

**Long-term Effects of the Gait Treatment Using a Wearable Cyborg Hybrid Assistive Limb in a Patient with Spinal and Bulbar Muscular Atrophy: A Case Report with 5 Years of Follow-Up**

**Kensuke Iijima¹, Hiroki Watanabe²*, Yuichi Nakashiro¹, Yuki Iida^3^, Michio Nonaka^3^**

**Fumio Moriwaka^3^, Shinsuke Hamada^3^**

*** Correspondence:**Hiroki Watanabe, PT, PhD

# E-mail: [watanabe.hiroki.gb@u.tsukuba.ac.jp](mailto:watanabe.hiroki.gb@u.tsukuba.ac.jp)

# Supplementary Figures and Tables

## Supplementary Figures


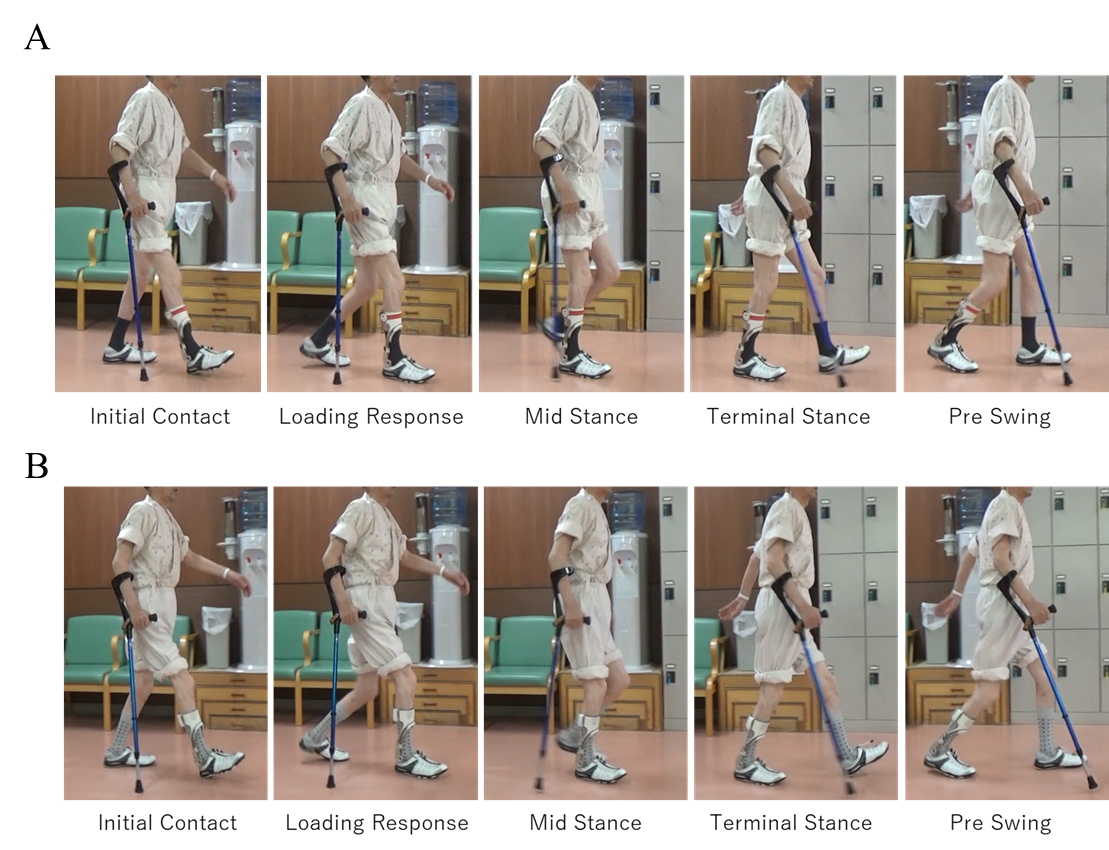


**Supplementary Figure S1.** Changes in gait posture before and after the HAL gait treatment during course 1

A: Gait posture before the gait treatment in course 1

B: Gait posture after the gait treatment in course 1

**Supplementary Figure 1.** The figure legends are required to have the same font as the main text, 12 point normal Times New Roman, single spaced. Please use a single paragraph for each legend and prepare the figures keeping in mind the PDF layout.
